# Identification and selection of reference genes for gene expression analysis by quantitative real-time PCR in *Suaeda glauca’*s response to salinity

**DOI:** 10.1038/s41598-021-88151-5

**Published:** 2021-04-21

**Authors:** Meng Wang, Tingting Ren, Prince Marowa, Haina Du, Zongchang Xu

**Affiliations:** 1 College of Agronomy, Qingdao Agricultural University, Qingdao, People’s Republic of China; 2 Marine Agriculture Research Center, Tobacco Research Institute of Chinese Academy of Agricultural Sciences, No. 11, Ke Yuan Jing 4th Road, Laoshan District, Qingdao, 266101 Shandong People’s Republic of China; 3 Crop Science Department, University of Zimbabwe, Harare, Zimbabwe

**Keywords:** Plant sciences, Plant stress responses, Salt

## Abstract

Quantitative real-time polymerase chain reaction (qPCR) using a stable reference gene is widely used for gene expression research. *Suaeda glauca* L. is a succulent halophyte and medicinal plant that is extensively used for phytoremediation and extraction of medicinal compounds. It thrives under high-salt conditions, which promote the accumulation of high-value secondary metabolites. However, a suitable reference gene has not been identified for gene expression standardization in *S. glauca* under saline conditions. Here, 10 candidate reference genes, *ACT7*, *ACT11*, *CCD1*, *TUA5*, *UPL1*, *PP2A*, *DREB1D*, *V-H*^+^*-ATPase*, *MPK6*, and *PHT4;5*, were selected from *S. glauca* transcriptome data. Five statistical algorithms (ΔCq, geNorm, NormFinder, BestKeeper, and RefFinder) were applied to determine the expression stabilities of these genes in 72 samples at different salt concentrations in different tissues. *PP2A* and *TUA5* were the most stable reference genes in different tissues and salt treatments, whereas *DREB1D* was the least stable. The two reference genes were sufficient to normalize gene expression across all sample sets. The suitability of identified reference genes was validated with *MYB* and *AP2* in germinating seeds of *S. glauca* exposed to different NaCl concentrations. Our study provides a foundational framework for standardizing qPCR analyses, enabling accurate gene expression profiling in *S. glauca*.

## Introduction

*Suaeda glauca* L. is a succulent halophyte that belongs to the Chenopodiaceae family^1^. It is widely distributed throughout Asia, particularly in the coastal areas of China^2^. *Suaeda glauca* is highly resistant to salt stress and thrives even in the presence of salt contents exceeding 0.48%, although no salt glands or vesicles are present in the leaves^1^. This plant is widely used for phytoremediation of saline soils primarily because of its Na^+^ hyperaccumulation and fast-growing properties as well as its ability to produce large amounts of biomass^3, 4^. *S. glauca* also shows potential for phytoremediation of Cd, Pb, and Mn in contaminated soils or mine tailings^5^ and is used as a traditional Chinese medicinal plant to treat fever and alleviate stagnation^6^. The abundance of secondary metabolites makes *S. glauca* a multi-purpose bio-resource for both ecological and health-related issues. Particularly, the ethyl acetate-soluble fraction of the methanol extract of *S. glauca* is reportedly hepatoprotective^7^. Additionally, the anti-inflammatory, anti-mutagenic, anti-oxidative, and other bioactive properties of the flavonoid gallic acid^8^ lead to protective effects in the liver^9^. However, the content of gallic acid in *S. glauca* ranges between 0.62 and 0.89%^10^; this low content of the active agent is the primary limitation of nearly all medicinal plants. Previous studies reported that in medicinal herbs, the contents of secondary metabolites including soluble phenolics, anthocyanins, and flavonoids were increased under salt stress^11, 12^. Thus, salinity may be used to boost the synthesis of secondary metabolites of interest in salt-tolerant medicinal plants. However, the specific molecular regulatory mechanism is poorly understood. Furthermore, many reports have indicated that the synthesis and accumulation of plant secondary metabolites are significantly correlated with the expression level of functional genes in their biosynthesis pathways^13, 14^. Recent research on increasing the production of active compounds in medicinal plants through recalibration and/or engineering of pivotal genes along the biosynthesis pathways has been reported ^15^. To enable fine-tuning of these processes, gene expression levels must be gauged using appropriate technical methods.

Northern blotting, semi-quantitative reverse transcription PCR, quantitative real-time polymerase chain reaction (qPCR), and in situ hybridization are routinely used to investigate gene expression^16^. Among them, qPCR is highly sensitive, rapid, reproducible, specific, and safe (does not use radioactive substances) and only requires a small amount of RNA^16–20^. Although qPCR has been widely used to evaluate the transcription levels of mRNA, obtaining accurate and reliable results is challenging. The process of RNA extraction and quality of RNA, reverse transcription efficiency, PCR, and even sample loading are critical factors potentially undermining the reliability and reproducibility of the results^21, 22^. To ensure the accuracy of gene expression data, endogenous genes are used as references to compare the expression levels of other genes^18^. The expression level of reference genes in different samples should be consistent and is unaffected by experimental conditions^17^. Glyceraldehyde 3-phosphate dehydrogenase (*GAPDH*), 18S ribosomal RNA (*18S rRNA*), ubiquitin-conjugating enzyme gene (*UBC*), β-tubulin (*β*-*TUB*), actin (*ACT*), α-tubulin (*α*-*TUB*), clathrin adaptor complex subunit (*CAC*), protein phosphatase 2 regulatory subunit A (*PP2A*), elongation factor 1-α (*EF1α*), polyubiquitin (*UBQ*), SAND gene family members (*SAND*), TIP41-like protein gene (*TIP41*), cyclophilin (*CYP*), and DnaJ-like protein gene (*DNAJ*) have been applied as reference genes for expression normalization in the halophyte plants *Salicornia europaea*^23^ and *S. aralocaspica*^24^ and medicinal herbs *Glycyrrhiza glabra*^25^ and *Apocynum venetum*^26^, *Actinidia chinensis*^27^, *Citrullus lanatus*^28^, and *Arachis hypogaea*^29^, among others. However, the reported reference genes are not commonly used in all plants, particularly under plant stress conditions^30, 31^. The ideal reference genes for evaluating salinity stress were found to be *CAC* and *UBC* in *S. europaea*, whereas *ACTIN* and *GAPDH* were used for drought stress^23^. Additionally, to evaluate salinity stress, the ideal reference genes were shown to be *ACTB* and *TUBB* in *Onchidium reevesii*^32^ and *TUBα* and *18S rRNA* in *Hibiscus cannabinus*^33^, suggesting that the reliability of reference genes is species-specific^34^. Therefore, for specific plants and/or specific experimental conditions, molecular screening and identification of internal reference genes is necessary. The selection and evaluation of reference genes in *S. glauca* remain unreported. Thus, identifying the most suitable reference genes for *S. glauca* can advance further gene expression analysis protocols, which are essential methods for revealing how genes are related to the plant physiology and molecular biology.

This study was conducted to identify, select, and validate optimal reference genes in *S. glauca* for normalizing gene expression levels via qPCR under salinity stress. A total of 10 candidate reference genes, *ACT7*, *ACT11*, *CCD1*, *TUA5*, *UPL1*, *PP2A*, *DREB1D*, *V-H*^+^*-ATPase*, *MPK6*, and *PHT4;5*, were screened, and the stability of their expression in different tissues of *S. glauca* exposed to different concentrations of NaCl was quantified using five statistical algorithms (ΔCq, geNorm, NormFinder, RefFinder and BestKeeper). Finally, the expression profiles of *MYB* and *AP2* were analyzed in germinating seeds of *S. glauca* exposed to different concentrations of NaCl to evaluate the usefulness of the selected reference genes.

## Results

### Primer specificity and amplification efficiency test of candidate reference genes

Standard PCR was used to verify the primer specificity in the amplification of all 13 candidate reference genes. Based on the results, *UBC28* and *TIM* showed multiple bands using cDNA as the template (Supplementary File S2A), whereas *EF1α* showed multiple bands amplified from both cDNA and genomic DNA (Supplementary File S2). These three genes were therefore discarded from further analysis. The other primers yielded a single and clear band of the expected size and there was no primer-dimer formation (Supplementary File S2). Further Sanger sequencing confirmed that the sequences of the amplified fragments were consistent with that given on NCBI, indicating the specificity of the primer pairs. The presence of a clear and single peak in melting curve analysis further verified the specificity of each primer set (Supplementary File S2). The details of the gene names, accession number, primer sequence, amplification length and efficiency, and correlation coefficient are shown in Table 1. The qPCR efficiency of all 10 candidate reference genes ranged from 87% (*MPK6*) to 119% (*ACT7*). The determination coefficients (R^2^) of the regression equation varied from 0.9763 for *MPK6* to 0.9994 for *UPL1*.

**Table 1:** Primer sequences and amplification characteristics of candidate reference genes of *S. glauca* used in this study.

Accession number	Arabidopsis ortholog locus	Identity (%)	Gene Name	Primer sequence 5′–3′ (F)	Amplicon length	Efficiency (%)	R^2^
BE859265.1	AT5G09810	0.86	ACT7	F: TAATCATCAAAATCCTGAGGA	103	119	0.9972
				R: ATTATGGTATGTAATCTTTGCGG			
MF893334.1	AT3G12110	0.81	ACT11	F: TGTTGCTCCAGAAGAGCATC	116	96	0.9969
				R: CATACATGGCAGGGACATTG			
BF114443.1	AT3G63520	0.85	CCD1	F: CCCAATCAAGGGTTCACTTC	108	107	0.9988
				R: GTGTTGAGGTTGTGAAGAATCA			
AW990992.1	AT5G19780	0.86	TUA5	F: GGCACAATGCACTAAGCAAC	110	98	0.9991
				R: AAGGTGCCGAGGATGATGAT			
BE240972.1	AT1G55860	0.84	UPL1	F: GAGTGGTACCAGCTATTGTC	103	114	0.9994
				R: AGACAGAATTAGGGTTTGGCT			
BE859200.1	AT1G64230	0.81	UBC28	F: CCCTCCAGATTATCCATTTAAG	106	–	–
				R: CTCCTTTAGGATGTCAAGACA			
BE644594.1	AT5G60390	0.91	EF1α	F: AGACCAACAAGTACTACTGCA	108	–	–
				R: CAATAATAAGGATAGCGCAGTC			
BE240909.1	AT1G69960	0.78	PP2A	F: CTTAGTATTCCCATTTCTTCATCT	144	104	0.9975
				R: ATGAGGACACAAAAAGAGCCAT			
KM679415.1	AT5G51990	0.71	DREB1D	F: CGACAGACACTAGGGAAATTC	107	95	0.9923
				R: GTCATTCATGCTGCTATTCTC			
BE231385.1	AT2G21170	0.77	TIM	F: GTTGTTACTATGGCTGGCTC	111	–	–
				R: CAATGTTGCACTGTTCAAGTCT			
BF145083.1	AT3G42050	0.77	V-H + -ATPase	F: CTAGCATAATTTCTGCAAAGCC	119	107	0.994
				R: CTGTTCCAGTGATCAACTTAGT			
BE656716.1	At2g43790	0.81	MPK6	F: CAACCTCATTCATCAGTCATCA	112	87	0.9763
				R: TGGTTTGCGGTGGTTGATTAG			
AW982148.1	AT5G20380	0.75	PHT4;5	F: AGCAACAGCATTCGTTCCAG	106	106	0.9983
				R: GATCTGTGGCAGCTGATGGT			
				R: AACTTCAAAGGCAATGTTGAAAAC			
BE644575.1	At5g52660	0.85	MYB	F: CTGCTAATGTGGTGTCACCAT	114	98	0.9923
				R: CATGCTCTTCTTCAGTCCAAC			
BE231371.1	–	–	AP2	F: AACTCTTTCTTCCTTAATCACTCT	124	95	0.9748
				R: CGTCGATAAAGTTCTCATTTTTAC			

Amplicon length was obtained using cDNA as a template.

### Expression profiling of candidate reference genes in different tissues under various salinity stresses

**Figure 1:**
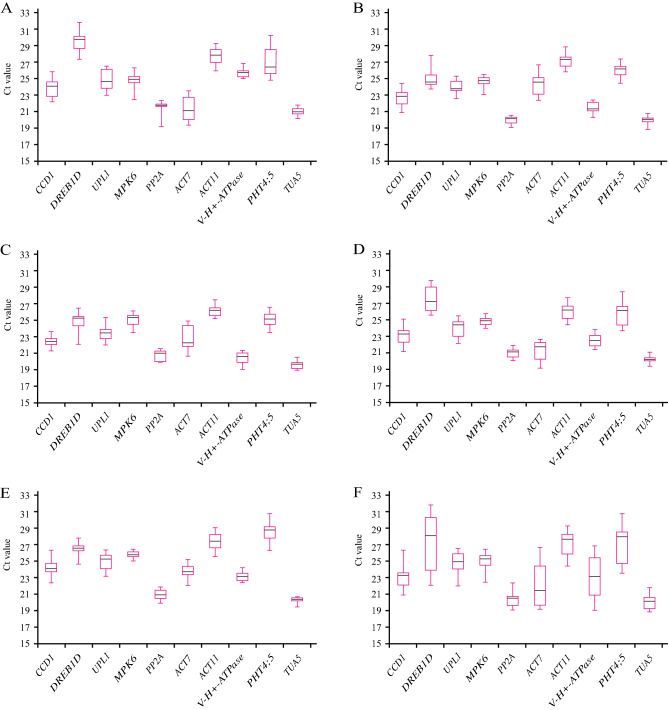
Boxplots showing the expression patterns of candidate reference genes of *S. glauca* represented by raw Cq values. The box indicates the 25th and 75th percentiles, and whisker caps represent the maximum and minimum values. The black line across the box depicts the median. (**A**) Thirteen tissue samples derived from 1/10 MS liquid medium treatment. (**B**) Thirteen tissue samples derived from 100 mM NaCl treatment. (**C**) Thirteen tissue samples derived from 300 mM NaCl treatment. (**D**) Fifteen germinating seed samples derived from NaCl gradient treatment. (**E**) Eighteen seedling samples derived from NaCl gradient treatment. (**F**) All 72 samples used in this study.

The cycle threshold (Cq) values generated from qPCR for all 10 candidate reference genes were obtained and analyzed, and their variation under each treatment is presented in Fig. 1.

The Cq values of these genes varied from 18.92 (*TUA5*, Fig. 1B,F) to 31.75 (*DREB1D*, Fig. 1A,F), suggesting that *TUA5* and *DREB1D* were the highest and least expressed genes, respectively. Candidate reference genes in different treatments showed a somewhat similar expression pattern. However, their expression levels differed under different treatments. While selecting the ideal reference genes among different tissues, *PP2A* showed the highest expression level (mean Cq of 20.91), and *DREB1D* exhibited the lowest expression level (mean Cq of 30.67) following treatment with only 1/10 MS liquid medium (Fig. 1A). The mean Cq values for *TUA5* and *ACT11* were 19.20 and 27.04, and 19.12 and 26.01, for samples obtained from plants exposed to 100 and 300 mM NaCl solution, respectively (Fig. 1B,C). Interestingly, the expression levels of *DREB1D* and *H*^+^*-ATPase* were markedly increased by NaCl solution treatment (Fig. 1A–C). *ACT7* (mean Cq of 21.10) and *TUA5* (mean Cq of 20.13) had the highest expression levels in the seed and seedling samples treated with different NaCl concentrations, respectively. However, *DREB1D* (mean Cq of 27.22) and *PHT4;5* (mean Cq of 28.93) showed the lowest expression levels in the seed and seedling samples treated with different NaCl concentrations, respectively (Fig. 1D,E). *TUA5*, *PP2A*, and *MPK6* showed relatively high expression levels with a relatively narrow range of ΔCq of 2.94, 3.32, and 4.00, respectively, in all samples, whereas *DREB1D*, *V-H*^+^*-ATPase*, *ACT7*, and *PHT4;5* showed relatively high variation with ΔCq values of 9.73, 7.82, 7.49, and 7.25, respectively (Fig. 1F).

### Stability assessment of candidate reference genes

Four specialized analytical tools, geNorm, NormFinder, BestKeeper, and RefFinder, were used to further assess the stability of the candidate reference genes in different responses of *S. glauca* tissues to salinity stress.

#### geNorm analysis

**Figure 2:**
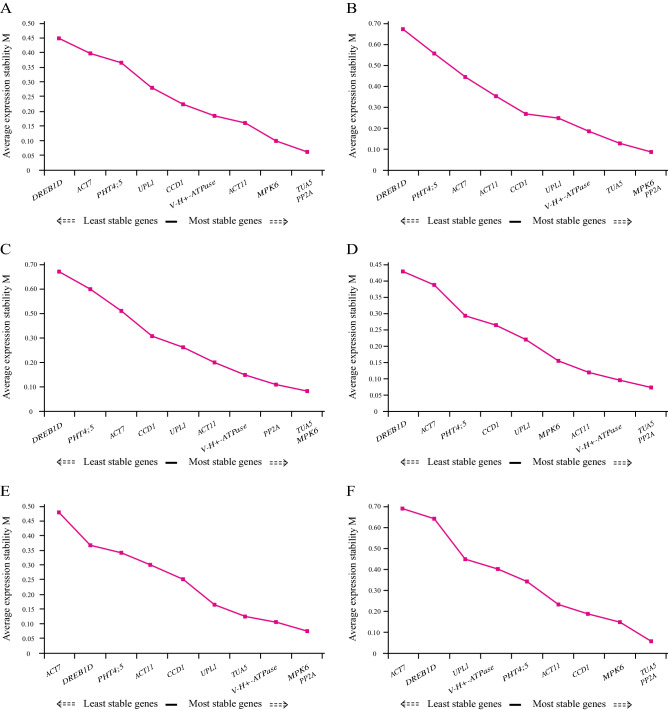
Gene expression stability values (*M*) of ten candidate reference genes validated via geNorm program. The least stable genes are on the left with higher *M*-values, and the most stable genes are on the right with lower *M*-values. (**A**) Thirteen tissue samples derived from 1/10 MS liquid medium treatment. (**B**) Thirteen tissue samples derived from 100 mM NaCl treatment. (**C**) Thirteen tissue samples derived from 300 mM NaCl treatment. (**D**) Fifteen germinating seed samples derived from NaCl gradient treatment. (**E**) Eighteen seedling samples derived from NaCl gradient treatment. (**F**) All 72 samples used in this study.

**Figure 3:**
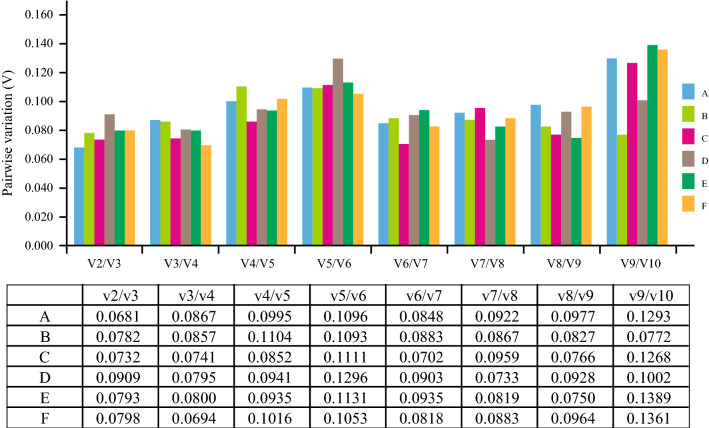
Optimal number of reference genes in different experimental groups calculated using geNorm. Pairwise variation (V_n_/V_n+1_) analysis of 10 candidate reference genes analyzed in six sample subsets. (**A**) Thirteen tissue samples derived from 1/10 MS liquid medium treatment. (**B**) Thirteen tissue samples derived from 100 mM NaCl treatment. (**C**) Thirteen tissue samples derived from 300 mM NaCl treatment. (**D**) Fifteen germinating seed samples derived from NaCl gradient treatment. (**E**) Eighteen seedling samples derived from NaCl gradient treatment. (**F**) All 72 samples used in this study.

In geNorm analysis, a lower M value indicates more stable gene expression and vice versa. All M values of the 10 candidate reference genes in each treatment were lower than 0.7 (Fig. 2), which is much lower than the default limit of 1.5, indicating high expression stability. However, for tissue experiments, the three most stable genes were *TUA5*, *PP2A*, and *MPK6* with the lowest *M* values, whereas *DREB1D* was the most unstable gene (Fig. 2A–C). In the seed group, *TUA5* and *PP2A* were the two most stable genes at all different concentrations of NaCl treatments (Fig. 2D). In the seedlings group, *PP2A* and *MPK6* were the most stable genes, followed by *V-H* + *-ATPase*, whereas *ACT7* and *DREB1D* were the least stable genes (Fig. 2E). Finally, for all sample sets, *TUA5* and *PP2A* showed the lowest *M* value, and *ACT7* was the most unstable gene (Fig. 2F). To determine the optimal number of reference genes required for accurate experimental normalization, pairwise variations of the normalization factor (V_n_/V_n+1_) were analyzed using geNorm. The V_2_/_3_ values of all different experimental groups were below the threshold value of 0.15, indicating that two reference genes are sufficient to normalize gene expression data (Fig. 3).

#### NormFinder analysis

NormFinder, which is an analysis program based on the variation estimation statistical approach, was used to evaluate the expression stability of the candidate reference genes. A lower average expression stability indicates more stable gene expression. The stability values of the candidate reference genes in each treatment are listed in Table 2. For tissues experiment under 1/10 MS liquid medium, *PP2A* and *TUA5* were the most stable reference genes and *DREB1D* was the least stable gene, which agreed with the results of geNorm analysis. For tissues experiment under 100 mM NaCl, *MPK6* and *PP2A* were the most stable reference genes, whereas *ACT11* was the least stable gene. Similarly, the most stable reference genes identified in tissue experiments under 300 mM NaCl were *TUA5* and *PP2A*, whereas *PHT4;5* was the least stable gene. The order of reference gene stability in seeds under NaCl treatments was: *MPK6* > *PP2A* > *TUA5* > *V-H*^+^*-ATPase* > *UPL1* > *CCD1* > *ACT11* > *ACT7* > *PHT4;5* > *DREB1D*. The ranking of gene stability across seedlings under NaCl treatment was: *PP2A* > *MPK6* > *TUA5* > *ACT11* > *CCD1* > *V-H*^+^*-ATPase* > *PHT4;5* > *ACT7* > *UPL1* > *DREB1D*. In all samples, *TUA5*, *PP2A*, and *MPK6* were the most stable reference genes and *DREB1D* and *PHT4;5* were the least ranked reference genes, which agrees with the results of geNorm analysis.

**Table 2:** Stability assessment of all candidate reference genes of *S. glauca* plants’ response to salinity stresses calculated using the NormFinder algorithms.

Order	A		B		C		D		E		F	
	Gene	Stability	Gene	Stability	Gene	Stability	Gene	Stability	Gene	Stability	Gene	Stability
1	*PP2A*	0.059	*MPK6*	0.152	*TUA5*	0.127	*MPK6*	0.092	*PP2A*	0.124	*TUA5*	0.069
2	*TUA5*	0.1	*PP2A*	0.153	*PP2A*	0.127	*PP2A*	0.106	*MPK6*	0.138	*PP2A*	0.113
3	*MPK6*	0.124	*TUA5*	0.168	*MPK6*	0.157	*TUA5*	0.126	*TUA5*	0.143	*MPK6*	0.143
4	*CCD1*	0.132	*UPL1*	0.189	*V-H*^+^*-ATPase*	0.173	*V-H*^+^*-ATPase*	0.200	*ACT11*	0.161	*ACT7*	0.192
5	*UPL1*	0.147	*CCD1*	0.207	*ACT11*	0.183	*UPL1*	0.205	*CCD1*	0.206	*V-H*^+^*-ATPase*	0.209
6	*V-H*^+^*-ATPase*	0.153	*V-H*^+^*-ATPase*	0.217	*CCD1*	0.185	*CCD1*	0.206	*V-H*^+^*-ATPase*	0.219	*CCD1*	0.227
7	*ACT11*	0.189	*PHT4;5*	0.252	*UPL1*	0.241	*ACT11*	0.218	*PHT4;5*	0.265	*UPL1*	0.230
8	*ACT7*	0.261	*ACT7*	0.271	*ACT7*	0.306	*ACT7*	0.304	*ACT7*	0.279	*DREB1D*	0.302
9	*PHT4;5*	0.301	*DREB1D*	0.372	*DREB1D*	0.356	*PHT4;5*	0.368	*UPL1*	0.302	*ACT11*	0.317
10	*DREB1D*	0.346	*ACT11*	0.394	*PHT4;5*	0.408	*DREB1D*	0.394	*DREB1D*	0.440	*PHT4;5*	0.353

A: Thirteen tissue samples derived from 1/10 MS liquid medium treatment.

B: Thirteen tissue samples derived from 100 mM NaCl treatment.

C: Thirteen tissue samples derived from 300 mM NaCl treatment.

D: Fifteen germinating seed samples derived from NaCl gradient treatment.

E: Eighteen seedling samples derived from NaCl gradient treatment.

F: All 72 samples used in this study.

#### BestKeeper analysis

The SD and *r* value of candidate reference genes in each treatment are listed in Table 3. Similar to the results of geNorm and NormFinder analysis, the three most stable genes were *MPK6*, *PP2A*, and *TUA5*, whereas *DREB1D* was the least stable gene in tissues (1/10 MS liquid medium). In addition to *PP2A*, *TUA5*, and *MPK6*, *V-H*^+^*-ATPase* was regarded as relatively stable in tissue experiments, both in 100 and 300 mM NaCl treatments. However, the two least stable genes in tissues after 100 mM NaCl treatment were *DREB1D* and *ACT7*. *DREB1D* and *PHT4;5* were the two least stable genes in 300 mM NaCl-treated tissues. For seed and seedling experiments, the top three most stable genes were again *PP2A*, *TUA5*, and *MPK6*, whereas *DREB1D* was the least stable gene for the two experiments. Overall, in the total samples, similar to the geNorm and NormFinder analyses, BestKeeper showed that *TUA5*, *PP2A*, and *MPK6* were the most stable genes and *PHT4;5* and *DREB1D* were the least stable genes.

**Table 3:** Stability assessment of all candidate reference genes of *S. glauca* plants’ response to salinity stresses calculated using the BestKeeper algorithms.

Order	A			B			C			D			E			F		
	Gene	SD	coeff. of corr. [*r*]	Gene	SD	coeff. of corr. [*r*]	Gene	SD	coeff. of corr. [*r*]	Gene	SD	coeff. of corr. [*r*]	Gene	SD	coeff. of corr. [*r*]	Gene	SD	coeff. of corr. [*r*]
1	*MPK6*	0.49	0.998	*PP2A*	0.53	0.998	*TUA5*	0.50	0.999	*PP2A*	0.53	0.999	*TUA5*	0.50	0.999	*MPK6*	0.58	0.999
2	*PP2A*	0.41	0.978	*V-H*^+^*-ATPase*	0.48	0.983	*PP2A*	0.44	0.980	*MPK6*	0.51	0.979	*PP2A*	0.50	0.979	*PP2A*	0.50	0.982
3	*TUA5*	0.45	0.971	*TUA5*	0.53	0.971	*V-H*^+^*-ATPase*	0.51	0.971	*TUA5*	0.48	0.978	*MPK6*	0.56	0.977	*TUA5*	0.53	0.979
4	*CCD1*	0.22	0.964	*MPK6*	0.30	0.966	*MPK6*	0.33	0.971	*V-H*^+^*-ATPase*	0.27	0.972	*V-H*^+^*-ATPase*	0.29	0.969	*UPL1*	0.23	0.970
5	*V-H*^+^*-ATPase*	0.38	0.961	*ACT11*	0.44	0.949	*CCD1*	0.46	0.966	*UPL1*	0.42	0.955	*ACT11*	0.46	0.967	*ACT11*	0.47	0.962
6	*ACT11*	0.52	0.881	*CCD1*	0.61	0.826	*ACT11*	0.60	0.874	*ACT11*	0.54	0.883	*UPL1*	0.54	0.839	*ACT7*	0.61	0.847
7	*UPL1*	0.30	0.76	*UPL1*	0.33	0.774	*UPL1*	0.41	0.729	*CCD1*	0.33	0.777	*CCD1*	0.31	0.756	*CCD1*	0.35	0.774
8	*PHT4;5*	0.53	0.718	*PHT4;5*	0.54	0.719	*ACT7*	0.64	0.723	*PHT4;5*	0.65	0.726	*DREB1D*	0.64	0.722	*PHT4;5*	0.63	0.627
9	*ACT7*	0.40	0.471	*ACT7*	0.49	0.472	*PHT4;5*	0.46	0.472	*DREB1D*	0.49	0.380	*ACT7*	0.49	0.278	*V-H*^+^*-ATPase*	0.43	0.472
10	*DREB1D*	0.28	0.083	*DREB1D*	0.30	0.187	*DREB1D*	0.40	0.289	*ACT7*	0.40	0.088	*PHT4;5*	0.28	0.085	*DREB1D*	0.36	0.193

A: Thirteen tissue samples derived from 1/10 MS liquid medium treatment.

B: Thirteen tissue samples derived from 100 mM NaCl treatment.

C: Thirteen tissue samples derived from 300 mM NaCl treatment.

D: Fifteen germinating seed samples derived from NaCl gradient treatment.

E: Eighteen seedling samples derived from NaCl gradient treatment.

F: All 72 samples used in this study.

#### RefFinder analysis

**Figure 4:**
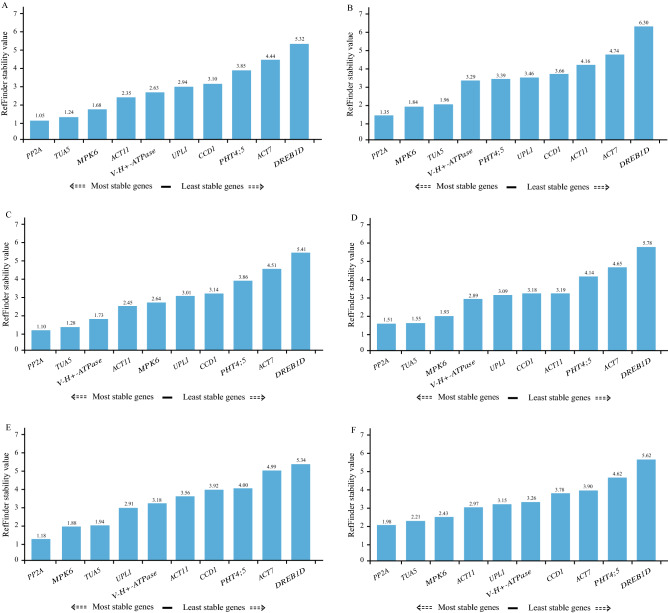
Expression stability of candidate genes in different tissues of *S. glauca* response to salinity validated by RefFinder program. (**A**) Thirteen tissue samples derived from 1/10 MS liquid medium treatment. (**B**) Thirteen tissue samples derived from 100 mM NaCl treatment. (**C**) Thirteen tissue samples derived from 300 mM NaCl treatment. (**D**) Fifteen germinating seed samples derived from NaCl gradient treatment. (**E**) Eighteen seedling samples derived from NaCl gradient treatment. (**F**) All 72 samples used in this study.

The comprehensive rankings of the candidate reference genes were ordered by the weighted geometric mean obtained by the RefFinder algorithm, which was based on the results of standard analysis programs (geNorm, NormFinder, BestKeeper). *PP2A* displayed the highest stability among all treatment samples, consistently ranking at the top in all subsets [tissues treated with 1/10 MS liquid medium, 100 mM NaCl, and 300 mM NaCl; germinating seeds under different NaCl concentrations; seedlings under different NaCl concentrations; and all samples] (Fig. 4). *TUA5* was the second-most stable gene, which consistently ranked in second place, in four experiments (tissues (1/10 MS liquid medium), tissues (300 mM NaCl), germinating seeds (NaCl gradient), and all samples) and third in two experiments (tissues (100 mM NaCl), and seedlings (NaCl gradient)) (Fig. 4). *MPK6* was followed by *TUA5*, consistently ranking third. However, the order of the least stable genes among all treatment samples was *DREB1D* > *ACT7* > *PHT4;5* (Fig. 4).

### Validation of identified stable reference genes

**Figure 5:**
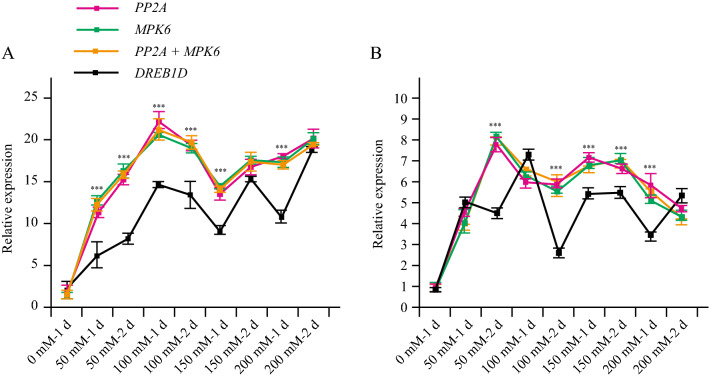
Relative expression levels of *MYB* (**A**), and *AP2* (**B**), normalized by different candidate reference genes, including the most or the least stable reference genes. The asterisks indicate significant differences from *DREB1D* using Student’s *t* test (P < 0.01).

To validate the suitability of the two selected reference genes, *PP2A* and *TUA5,* and the least stable reference gene *DREB1D*, the salt-induced expression level of *MYB* and *AP2* in germinating seeds was normalized via qPCR. *MYB* (Fig. 5A) and *AP2* (Fig. 5B) showed similar expression levels when a single or a combination of reference genes (*PP2A* and *TUA5*) was used to normalize the expression. The expression levels of *MYB* and *AP2* were upregulated in all seed samples treated with different concentrations of NaCl. However, when *DREB1D* (unstable gene) was used for normalization, the relative expression patterns of *MYB* and *AP2* differed as compared to the relative expression values obtained using the two most stable reference genes (*PP2A* and *TUA5*) (Fig. 5), highlighting the significance of selecting appropriate internal reference genes under salt stress.

## Discussion

Gene expression analysis is a primary method for revealing the mechanism of plants' responses to different conditions, including abiotic stresses ^23^. qPCR is an efficient and reliable method for assessing gene expression levels. However, the accuracy of the results is affected by the expression level and stability of internal reference genes. Using inappropriate reference genes can lead to misleading results ^42^. Hence, application of steadily expressed internal reference genes is key to obtaining accurate results ^20, 43^. Commonly used methods for selecting candidate internal reference genes include searching the literature or using housekeeping genes, such as genes involved in cell metabolism, glycolysis, protein degradation, and synthesis ^44^. However, previous studies reported that the expression level of internal reference genes obtained by this method may be unstable under different experimental conditions ^45^, greatly affecting the accuracy of the results. Compared with the validated reference genes, the expression level of target genes can show 100-fold deviation using inadequately validated reference genes. Thus, using reference genes obtained by traditional methods may lead to erroneous or even contradictory results ^21, 43^. Therefore, constant expression in different tissues or experimental treatments is a vital feature of an ideal reference gene ^46^. Generally, candidate reference genes belong to different types of functional genes, which can significantly reduce the risk of gene expression being affected. *S. glauca,* a succulent halophyte ^1^, is used as a traditional Chinese medicinal herb because of its health-promoting pharmacodynamic effects ^6^. In a previous study, *ACTIN* was used as the reference gene to normalize the salt response gene under salt stress ^2^. Similarly, *EF1α* was used as a reference gene in another *Suaeda* species (*S. salsa*) to normalize the gene expression level under salt stress ^47^. However, there are no reports on the systematic screening and identification of internal reference genes of *S. glauca* under salt stress. In this study, after a series of systematic screening steps, 10 reference genes were selected for further screening and validation as potential gene expression normalization genes under salt stress in *S*. *glauca*. Further assessment with ΔCq, geNorm, NormFinder, BestKeeper, and RefFinder software identified *PP2A* and *TUA5* as the two most stable reference genes, whereas *DREB1D* was the most unstable gene in all samples. Notably, these reference genes belong to different functional gene classes. A previous study reported that *GAPDH*, *ACTB*, and 18S and 28S rRNA were the most frequently used reference genes in articles published by high-impact journals (more than 90% cases) ^48^. However, in this study, none of these genes were identified as suitable reference genes for *S. glauca*. *GAPDH* was reported as the most appropriate reference gene under different salinity stress conditions for *S. aralocaspica*, which belongs to the same genus as *S. glauca* ^24^. The constitutively expressed, housekeeping enzyme GAPDH converts glyceraldehyde-3-phosphate into 1,3-bisphosphoglycerate, which plays a key role in glycolysis. Hence, *GAPDH* is widely used as a reference gene ^49^. Additionally, *AvGAPDH-2* has been identified as the most stable reference gene in another traditional Chinese herb, *A. venetum*, under salinity stress ^26^. Previous studies reported that *GAPDH* was one of the best reference genes in different tissue and organ samples of teak ^50^ and *Dendrocalamus latiflorus *^34^ because of its high stable expression. However, *GAPDH* was not a suitable reference gene in rice under water-deficient conditions because of the high expression instability ^51^. These results indicate that reference genes effective in one species may not be appliable in other species ^34^. Although orthologous genes of *DREB1D*, *PHT4;5*, and *ACT7* have been effectively used in studies of *Fagopyrum tataricum* ^52^, *A. hypogaea* ^29^, and *A. venetum* ^26^, our comprehensive analysis showed that these were the top three most unstable genes in *S. glauca*. In contrast, *PP2A* and *TUA5* were identified as the two most stable reference genes in the response of *S. glauca* to salinity. These genes were also chosen as optimal reference genes in *C. lanatus* ^28^, *A. hypogaea* ^29^, *Heterosigma akashiwo* ^53^ and *Eucalyptus* ^54^. In fact, many studies selected a series of reference genes that have been reported in other plants and screened them under various experimental conditions ^55^, which showed that commonly used reference genes exhibited universality in different plants. However, every gene chosen in this method must be verified before it can be used in a new plant. A universally applicable reference gene does not exist ^45, 56^.

Many previous studies reported that a single reference gene was not adequate for normalization. The expression results would generate 3–6.4-fold error using only one internal reference gene^21^. To obtain accurate results, two or more stable reference genes are considered as necessary^26, 57^. The geNorm program was used to determine the optimal number of internal reference genes for normalization with the pairwise variation parameters^21^. The result of pairwise variation analysis of all samples in this study revealed that the V2/3 value was below the threshold of 0.15^21^. Therefore, the combination of the most stable two internal reference genes would guarantee an accurate result. Transcription factors *MYB*^40^ and *AP2*^41^ are involved in the salt stress response. A previous study reported that the most responsive transcription factor families were AP2/ERF and MYB of *Lotus japonicus* treated with NaCl^58^. Hence, these salt-response genes were used to validate the suitability of the candidate reference genes in germinating seeds exposed to different NaCl concentrations. As shown in Fig. 5, both *MYB* and *AP2* were upregulated in all seed samples treated with NaCl when a single or a combination of reference genes (*PP2A* and *TUA5*) was used. The results are consistent with the findings that *MYB* and *AP2* are induced by salt stress. However, compared to using a single reference gene, the expression profiles of *AP2* and *MYB* were more accurate when a combination of reference genes (*PP2A* and *TUA5*) was used to normalize the expression (Fig. 5). Overall, our results revealed the most relevant reference genes for normalization of relative gene expression of *S. glauca* tissues and response to salinity stresses.

## Methods

### Plant material and salinity stress treatments

*Suaeda glauca* seeds were collected from a saline field in Dongying City, Shandong Province, China. All experimental procedures including investigation and collection were in accordance with local and national regulations. The voucher specimen of *S*. *glauca* has been deposited in Naturalis Biodiversity Center (herbarium ID L.1678537_544178685). Professor Jie Song (College of Life Science, Shandong Normal University, China) undertook the formal identification of *S*.*glauca* used in your study. We were allowed to collect the plant by the local government. Seeds of *S*.*glauca* were grown in a greenhouse with a temperature range of 25 °C ± 1 °C and 16-/8-h light/dark photoperiod cycles. The plants were grown in vermiculite and watered using 1/10 Murashige and Skoog (MS) liquid medium. Considering that *S. glauca* is autochthonous of saline landscapes and that the salt content and types of salts present in nature may differ from those of artificial setup, three groups of experiments were designed for tissue collection. A total of 60 four-week-old seedlings were sorted into three groups of 20 plants. One group was irrigated with 1/10 MS liquid medium, whereas the other two were irrigated with either 100 or 300 mM NaCl dissolved in 1/10 MS liquid medium. For each group, the following samples (roots, stems, leaves, and shoots) collected at different growth stages (seedlings, adult plants, and senescent plants), and followers were used for expression analyses of candidate reference genes screening. Samples for each tissue were collected from at least five individual plants and quickly frozen in liquid nitrogen and stored at − 80 °C throughout the sampling period. A series of NaCl solutions (0, 50, 100, 150, and 200 mM NaCl dissolved in 1/10 MS liquid medium) was used to treat the germinating seeds. Seed samples were collected at 1, 2, and 4 days after treatment. Furthermore, two-week-old seedlings were irrigated with a series of NaCl solutions (0, 50, 100, 150, and 200, and 400 mM NaCl dissolved in 1/10 MS liquid medium). The total plant samples were collected at 2, 4, and 8 days after treatment. All samples were stored at − 80 °C until further analysis.

### Total RNA extraction and cDNA synthesis

Total RNA was extracted from all samples using a MiniBEST Plant RNA Extraction Kit (code no. 9769; Takara, Shiga, Japan) according to the manufacturer’s instructions. As part of the extraction protocol, potential DNA contamination was digested with DNase I. The integrity of RNA was assessed by 1.2% agarose gel electrophoresis. Two complete and clearly defined RNA bands (28S and 18S) indicated that the extraction was successful. A NanoDrop 2000 Spectrophotometer (Thermo Fisher Scientific, Waltham, MA, USA) was used to determine RNA concentration and purity. The absorbance ratios at 260/280 and 260/230 nm of the RNA samples ranged from 1.8 to 2.0 and 2.0 to 2.4, respectively, indicating a suitable quality for subsequent analysis. According to the kit instructions [HiScript Q RT SuperMix for qPCR (+ gDNA wiper) Kit; Vazyme, Nanjing, China], 1 μg purified RNA extracted from each sample was reverse-transcribed into first-strand cDNA. Next, 20 μL cDNA products were diluted to a volume of 200 μL for qPCR. A mixture of an equal volume from a cDNA pool of all samples was used as the template to amplify the candidate *S. glauca* reference genes to examine the specificity and efficiency of our qPCR primer pairs.

### Candidate reference gene selection and primer design

Reference genes should be unaffected by experimental conditions and constitutively expressed, indicating their relatively stability^17^. However, reported reference genes are not commonly used in all plants, particularly under plant stress conditions^30, 31^. Hence, based on several published studies^23, 25–28, 32, 33, 35^, *ACT7*, *ACT11*, *CCD1*, *TUA5*, *UPL1*, *UBC28*, *EF1α*, *PP2A*, *DREB1D*, *TIM*, *V-H*^+^*-ATPase*, *MPK6*, and *PHT4;5,* which have been frequently reported. were selected as candidate reference genes in *S. glauca*. The coding sequences of these genes were obtained from GenBank according to an earlier transcriptome sequencing study^2^ and are listed in Supplementary File S1. The qPCR primers were designed with Primer 3 software. The length and Tm value of the primers for qPCR were identified with the OligoCalc tool^36^. Before qPCR, the PCR products of all primer pairs were electrophoresed on a 2% agarose gel stained with ethidium bromide to verify successful amplification and primer specificity. Primer specificity was validated via melting curve analyses following amplification using qPCR.

### qPCR conditions and amplification efficiency test

The 7500 Real-Time PCR system (Applied Biosystems, Foster City, CA, USA) was used to perform qPCR under the following conditions: 50 °C for 2 min and initial denaturation at 95 °C for 5 min, followed by 40 cycles at 95 °C for 10 s and 60 °C for 35 s. The dissociation curve was analyzed to determine the specificity of primer amplification, with the dissociation temperature ranging from 60 to 95 °C, by increasing the temperature stepwise by 1 °C/min^26^. The volume of the qPCR system was 20 μL. The reaction mixture consisted of 10 μL 2 × SYBR green mix (Q321, Vazyme), 0.4 μL ROX Reference Dye, 1 μL cDNA, and 0.4 μL of each forward and reverse primer (10 mM). Each sample was evaluated with three biological replicates. To identify the specific PCR amplification efficiency of the reference gene primer pairs, a tenfold dilution series (10–1000-fold dilution) of a cDNA isovolumetric mixing pool was used as templates for qPCR. The primer amplification efficiency (E) and correlation coefficient (R^2^) were calculated from the raw Cq values using the equation: E = 10^–1/slope^ – 1^28^, where the slope is derived from the regression equation, calculated using Excel linear regression parameter (Microsoft Corp., Redmond, WA, USA).

### Stability assessment of candidate genes

The statistical algorithm software programs geNorm^21^, NormFinder^22^, BestKeeper^37^, and RefFinder^38^ were used to analyze the expression stability of candidate reference genes.

The raw Cq value was used as input data for further analysis with RefFinder and BestKeeper. 2^−ΔCq^ value and 2^Cq^ value were used as input data for further analysis with geNorm and NormFinder, respectively. The parameters calculated using geNorm were the average gene expression stability measure (M) and average pairwise variation (V). A lower M value of the reference gene indicated more stable expression^21^. The parameter calculated by NormFinder was the stability value, which is related to the systematic error of each candidate gene. Smaller values indicate more stable gene expression^22^. The parameters calculated using BestKeeper were the SD values of Cq and coefficient of correlation (*r*) value. A gene with an SD value below 1.0 and *r* value close to one indicate more stable gene expression^37^. RefFinder generated a final comprehensive ranking of the most consistent and appropriate candidate reference genes according to the weighted geometric mean of the results of geNorm, NormFinder, and BestKeeper^38, 39^. The lowest geometric mean indicated the most stable gene.

### Validation of reference genes

To validate the reliability of the selected reference genes, the relative expression levels of two transcription factors *MYB*^40^ and *AP2*^41^, which are involved in the salt stress response, were used as stress indicator genes to validate the suitability of candidate reference genes using qPCR at germinating seed samples.

## Conclusion

We systematically evaluated the expression stability of different potential reference genes for qPCR in *S. glauca*. Thirteen typical reference genes were selected to identify the most stable reference genes for qPCR normalization in *S. glauca*. Based on the primer specificity results derived from the PCR results, *UBC28*, *TIM*, and *EF1α* were discarded because multiple bands were amplified from the cDNA and gDNA templates. *ACT7*, *ACT11*, *CCD1*, *TUA5*, *UPL1*, *PP2A*, *DREB1D*, *V-H*^+^*-ATPase*, *MPK6*, and *PHT4;5* were further analyzed in different tissues and under various salt concentration treatments. *PP2A* and *TUA5* were identified as the best reference genes, according to calculations performed using the ΔCq, geNorm, NormFinder, BestKeeper, and RefFinder programs. The reference genes were validated to profile the expression of *MYB* and *AP2* in germinating seeds exposed to NaCl. The present study identified appropriate reference genes for normalization of reliable qPCR data in different *S. glauca* tissues and under salinity stress, which can guide the determination of expression profiles of target genes in *S. glauca* plant and related species.

## Supplementary Information

Supplementary Information
