# Supplementary material for: Identification and selection of reference genes for gene expression analysis by quantitative real-time PCR in Suaeda glauca’s response to salinity
Source: Sci Rep. 2021 Apr 21;11:8569. doi: 10.1038/s41598-021-88151-5 (PMC8060425; doi:10.1038/s41598-021-88151-5)
Supplement: Supplementary file 1 — Supplementary Information [file 41598_2021_88151_MOESM1_ESM.docx]

# Identification and selection of reference genes for gene expression analysis by quantitative real-time PCR in *Suaeda glauca’*s response to salinity

**Meng Wang^1^, Tingting Ren^2^, Prince Marowa^3^, Haina Du^1^ and Zongchang Xu^2,*^**

^1^ College of Agronomy, Qingdao Agricultural University, Qingdao, China

^2^ Marine Agriculture Research Center, Chinese Academy of Agricultural Sciences, Qingdao, China

^3^ Crop Science Department, University of Zimbabwe, Harare, Zimbabwe.

**^*^**Corresponding Author: Zongchang Xu

Marine Agriculture Research Center, Chinese Academy of Agricultural Sciences, No. 11, Ke Yuan Jing 4^th^ Road, Laoshan District, Qingdao, 266101, Shandong, PR China

Email address: [xuzc1110@163.com](mailto:xuzc1110@163.com)

## Supplementary File 1

**Supplementary File 1: Partial mRNA sequences of candidate reference genes used in this study.**

>BE859265.1 ACT7

GAGAGAACTAGTCTCGATTTTTTTTTTTTTTTTTTTGAAATACAGACTGTCATTTTTTTTTTTCATTTTCCATGAACATAGAGATATATGGCAAAAAAACAGAGCCACATAATCATCAAAATCCTGAGGATTAACAAGGNGTGACCCAGAAAAATAGTAGAGTATAGAAATAAAACATTCTGCAATCCGCAAAGATTACATACCATAATTCTCGAAAAATAGTTACAACAAACAATAGAAATTCCAAGCTTTGGTCCCAAAAAAAACCACAAGCCCCTAAACCCTCTCCATCTCTCTCGACCAAATCAAATCAAATCATACACCACACTAATAGTTTTTTTTTTGACCAAACATAAAGAAAAACAAAGAGACAACAGGAGACTATGTAGGACTACAGATCTCAAAACTTATCTTCTTAGAAGCACTTTCGGTGAACAATTGATGGGCCAGATTCGTCGTACTCGCCCTTGGAGATCCACATCTGTTGGAAGGTGCTGAGTGATGCAAGGATAGACCCTCCTATCCAGACACTGTATTTCCTCTCGGGTGG

>MF893334.1 ACT11

ATGGCTGATGCAGAGGACATTCAACCTCTTGTCTGTGATAATGGAACTGGAATGGTCAAGGCTGGGTTTGCTGGAGACGATGCACCTAGAGCAGTCTTCCCTAGTATTGTTGGTCGTCCCCGACACACAGGTGTCATGGTTGGTATGGGCCAAAAGGATGCATATGTTGGAGATGAAGCTCAATCAAAGAGAGGTATTCTGACGTTGAAGTACCCAATTGAACATGGTATTGTTAGCAACTGGGATGACATGGAGAAGATCTGGCACCACACTTTCTATAATGAACTCCGTGTTGCTCCAGAAGAGCATCCTGTCTTGCTGACTGAGGCCCCGCTGAACCCCAAGGCTAACAGGGAGAAAATGACTCAGATTATGTTCGAGACATTCAATGTCCCTGCCATGTATGTTGCCATTCAAGCTGTTCTCTCATTGTATGCTAGTGGTCGTACTACAGGTATTGTACTCGATTCTGGTGATGGTGTAAGCCACACTGTGCCAATCTATGAAGGTTATGCTCTGCCCCATGCAATACTCCGTCTGGATCTTGCGGGTCGTGATCTCACAGATTATCTCATGAAAATCCTCACCGAGAGAGGTTACATGTTCACA

>BF114443.1 CCD1

TTGAGAAAATGGGTGTTGCAGAAGAAGGGAAGAAACACAATGAAGACAACAACTTGGAAACCAAAAATGGTGAAAAACAAGGNAATTGTTCATATTAACCCTAAACCCAATCAAGGGTTCACTTCAAAAGCAATTGATTGGTTGGAGTAAAATCATTGTGAAGTTGATGTATGATTCTTCACAACCTCAACACTATCTTTCTGGAAATTTTGCTCCTGTTGTTGATGAAACTCCTCCTGCTAAAGACTTGATTGTTCATGGACATCTCCCTGAATGCTTGAATGGGGAGTTTGTCCGTGTTGGACCCAATCCTAAGTTTGCTCCTGTGGCTGGGTATCACTGGTTTGATGGAGATGGGATGA

>AW990992.1 TUA5

CCTCGGGCCGAATTTTTTTTTATGAAATCTTGCAATTATGTTTTACACAAGACAGATAGAACACACAAAATACTGCACTTCAAGAAACCATCACCATTTCAAAGACAGAAATAATCCATGGAAACAGAAACAACTACATAGTTCTCAACCAAAAAGCTTGCACAAGACAATGGCACAATGCACTAAGCAACATAACCCAACTGGATACTTCTCAACAACCACAGNTAGACATAATCTTAGTACTCGCCGTGCGACATCACCATCATCATCCTCGGCACCTTCAGCACCAACTTCCTCATAGTCCTTCTCCAGAGCGGCAAGATCCTCACGAGCCTCANAGAATTCACCTTCCTCCATACCCTCACCAACATACCAGNGGACGAAAGCTCTCTTGGCATACATGAGGCCAAACTTGTGGTCAATCC

>BE240972.1 UPL1

TGCGAATGAGGTCAAATCAGGACCTGAAGGGGAGATTGAACGTCCAGTTTCAAGGTGAAGAAGGTATTGATGCTGGTGGCCTCACTAGAGAGTGGTACCAGCTATTGTCGAGGGTCATCTTTGATAAGGGAGCTCTACTCTTTACGACAGTTGGGAATAATGCGACCTTCCAGCCAAACCCTAATTCTGTCTATCAGACTGAACATCTTTCTTACTTCAAATTTGTTGGTCGTGTGGTTGCAAAGGCCTTATTCGATGGGCAGCTTTTGGATGTTTACTTCACCCGTTCCTTCTACAAGCATATTTTGGGAGTGAAGGTGACCTATCATGATATTGAGGCTG

>BE859200.1 UBC28

GCTTCCTATGGCTTCCAAACGAATTTTGAAGGAGCTCAAAGATCTCCAGAAAGATCCTCCTACTTCTTGCAGTGCTGGCCCTGTTGCTGAAGACATGTTTCATTGGCAAGCTACTATAATGGGTCCTACAGAAAGCCCGTATACTGGGGGAGTGTTTTTAGTGACAATTCATTTCCCTCCAGATTATCCATTTAAGCCACCCAAGGTTGCCTTCAGGACAAAAGTTTTCCACCCAAACATCAACAGCAATGGTAGTATATGTCTTGACATCCTAAAGGAGCAGTGGAGTCCTGCATTGACTATATCTAAGGTGTTGCTTTCAATATGTTCCTTGTTGACAGATCCAAACCCTGATGACCCCCTAGTGCCAGAGATCGCCCACATGTACAAGACTGACCGCTCAAAGTATGAGACAACCGCAAGGAGCTGGACGCANAAGTATGCTATGGGGTAAG

>BE644594.1 EF1α

CCGGTCACTTGATCTACAAGCTTGGTGGTATTGACAAGCGTGTGATCGAGAGATTCGAGAAGGAAGCTGCTGAGATGAACAAGAGGTCCTTCAAGTATGCATGGGTTCTCGACAAACTTAAGGCAGAGCGTGAACGTGGTATTACCATTGATATTGCTTTGTGGAAGTTTGAGACCAACAAGTACTACTGCACTGTCATTGATGCCCCTGGTCATCGTGACTTTATCAAGAACATGATTACTGGTACCTCTCAAGCTGACTGCGCTATCCTTATTATTGATTCCACCACTGGAGGTTTTGAGGCTGGTATCTCCAAGGATGGTCAGACCCGTGAGCACGCCCTTCTTGCTTTTACTCTCGGTGTCAAGCAAATGATTTGCTGNTGGAACAAGATGGATGCCACCACACCCAAGTACTCCAAGGCTAGGGACGATGAAATCGNGAAGGAAGNTTCATCATACATNAAGAAGGGTGGNTACAACCCANACAAG

>BE240909.1 PP2A

GAACTAGTCTCGAGTTTTTTTTTTTTTTTTTTTTTTAATTCACAATTCTGTACTGAATATATTAAGCCAAAAAAATGTGGATATGTAAACTGAGACAGTAGTTCCAACTCAGACAGCAAATTCAAAAACAACATCAACTTAAAAGGTATTCCCAATAAGCTATCATTCGATAAATGCCAAACTGAAGGCGACACTAGACCAAAAAAAAAAAAACTGCACAGATAATATAGAAAGAACTAAAGACTTAGTATTCCCATTTCTTCATCTCCATGCCATCTGGAGGAGTTCCCTCAACCTTCTTCGAGCCCACATCTTTCCAGTTTGTTGACAGCACTGTTCCATTCGACTCAACAAAGGATTTCTGCATGGCTCTTTTTGTGTCCTCATCGGCATCTCGATATATGTCTTGGAAAAACTTGTTCAAAGCTGCATCACCATCCAATTTTTCATCTTTTTCCTCTTTCTT

>KM679415.1 DREB1D

TACAGGGGAGTGAGGCAGAGGAATGGGAATAGGTGGGTGTGTGAGGTGCGTCCCCCTAATAACAAGTCTAGGATATGGCTTGGGACGTATCCTACAGCTGAGATGGCAGCGCGGGCACATGATGTGGCTGCATTGGCACTTAGAGGTAGGACAGCATGTCTAAACTTTGCTGACTCGGCATGGAGGCTGCGTGTCCCTGAATCGACAGACACTAGGGAAATTCAGAGAGCAGCAGCCGAGGCAGCAGAAGCATTTAGAGGGGAAGAAGAATCACAGGTAAATGCTGGGGAGAATAGCAGCATGAATGACAATGAAATGGAAATTACTACAGGTGAGAATGAGCTAGTAATGAATAGTAACCAAGAATTTTGGCAGGGAAATGCAGAATACATGGATGAGGAGGCAATGTTTGACATGCCGGGTTTACTAGCTAACATGGCCGAAGGATTGTTACTACCTCCGCCTTGTCCATATGGCGGATTCAGCTGGGATGACTCAGACAGTGACTATGAAGTCTCACTGTGGAGTTTCTCAGCGTAAAAAAATACAGGGAGATAGCATGATTAAGTTAGTTTCCATGCAGGTGCTTGGGGTGCATAGGCAGCATTGTTATTTTCCTTTTACCCTATTCATGCCAAAATATTTACAAAGGCATGGAATTTTAAGCAAGATTATTTGTATTAAAAAAATTCATTCCTAGAGGGTAGAGTAGAACGAAATTTCATCAGCGAAAATCGGAATTTTAAAAGGTAGAACTCAGAGAGTGACTACCACAGAAGATTTTCTGTGTGGCGACGAAAAAAAAAAAAAAAAAAA

>BE231385.1 TIM

ATCACTCTCAAAAAAAGGCGAAAGCATTCACACTTTTCACAGAAGAACAGTGAGGAGAGAGAAAATCCAAGAGCGTCGAAAATGGCGGTTGTATCAACTTCATTAGCGTCGCAACTAAGTAACCCTAACGCTGTTGTTTCAACTCAGTTTTCTGGTCTTCGACGATCCTTTCTCAAGTTTGAAACTAGCTCTTCTTCTTCAAATGAATGCTTTTTCCTGAATGTTGATTCTCATTTGCTCTTATCGTCTTCTCGCCGTGGTTCTAGAGGTGTTGTTACTATGGCTGGCTCTGGAAAGTTCTTCGTTGGCGGCAATTGGAAGTGTAATGGAACCAAAGAATCAATCACTAAGCTTGTTTCAGACTTGAACAGTGCAACATTGGAGGCTGATGTTGATGTTGTTGTCGCACCTCCGTTTGTTTACATTGATCAAGTTAAGAATTCTCTAACCAGTCAAGTAGAGATATCAGCACAGACTGGTGGATTGGAAAAGGTGGGGCTTTTACTGGAGAAATCAAGTGCTGAGCAATTGAAGGATCTTGGCTGCCAGTGGGTTATTCTT

>BF145083.1 V-H^+^-ATPase

TCTAAACTTTCAATTTAACACATTCATTTGTTCATTAATCTGTGTTTTTCTTCTTTGAAGTTCACAATTCTAGCATAATTTCTGCAAAGCCATGGCGACAGATCAAGCCGAACTCACCACTGATCAGGTTCTCAAAAGGGACATTCCATGGGAGACATACATGACAACTAAGTTGATCACTGGAACAGCTCTCCAACTTTTAAGGCGTTATGATAAAAGACCTGAGAGCCAAAGAGCATCATTGCTGGATGATGATGGTCCTTCATATGTTCGAGTGTTTGTGAGCATTTTACGTGATATATTCAAAGAAGAGACAGTGGAATATGTCCTTGCTTTAATCGATGAAATGCTCACTGCGAATCCCAAAA

>BE656716.1 MPK6

GAGAGAGAGAACTAGTCTCGAGTTTTTTCCATTTCTAGGGTTTCTTCAATTTGGAAGAAGAGAGCTCAAGCTCAAGCTTCAACCTCATTCATCAGTCATCACCACAACAATGGATGCTTCAAATGTTCTATCTTCCGACTCTGAGATGTCGGAAGTCGTTAATCCAACGCCTAATCAACCACCGCAAACCACCGATCACACTCATCAGCAACCACCGCAACAACAACAACAGCAACAAGTAAGTATTCCTGCAATTCAAAGCCATGGTGGAAAGTTCATCCAATACAACATATTTGGAAACGTGTTTGAGGTTACTTCTAAGTACAAACCTCCTATCATGCCTATTGGAAAAGGCGCTTATGGAATTGTTTGTTCGGCGTTGAATTCGGA

>AW982148.1 PHT4;5

CCTCGTGCCGAATTCGGCACGAGGGTCAGTTGCCGGGCGGATGGCTTGCCAAAATATATGGAGGCAGGCAAGTTCTAGCGTTAGGAGTCTTGGTTTGGTCATTAGCAACAGCATTCGTTCCAGTTCTAGCTGGATATATGCCAGGACAATTTTGTCAAGAATTTTGGTTGGAATAGGTGAAGGTGTTTCACCATCAGCTGCCACAGATCTTATTGCCAGATCTATACCTTTGGAGGAGAGGTCACGAGCCGTTGCATTTGTTTTTGGTGGTTTAAGTGTAGGAAGCGTAGCAGGGCTTCTCTTGGCTCCTCGACTCATTCAGAATTTTGGCTGGGAATATGTATTTTACTTATTTGGCCTCGTGGGTGTAGTATGTAGGCTTTCACTTGCCAATGTCAAGCCTGACTGAAGTATGACTTTGATACCCCCAAAAAAACCTTGAAGGTTTTTGGGCTTTCAG

>BE644575.1 MYB

GTAGAAGCTTTTTTCATAGGTAAAACATAAAAAGATTTAAAATTTATAAATTTGGTGATTACCCCAGAATTGAATTCTGTGGTTTGGGATGAATCAAGGTCAATCAAATAGTTCTGCTTCTGCAGCTGCTAATGTGGTGTCACCATCATCAGCTGCAGGAGAAGGGTCTTCAGGGAAGAAAGTTAGGAAGCCTTATACAATTACTAAGTCTCGTGAAAGTTGGACTGAAGAAGAGCATGATAAATTCCTTGAAGCACTTCAATTGTTCGATCGTGATTGGAAGAAGATTGAAGATTTTGTTGGCTCAAAAACTGTGATTCAGATTCGCAGTCATGCTCAGAAATACTTTTTGAAAGNTCAAGAAAA

>BE231371.1 AP2

CTTACTTAAACCCCCCTCTTTTCGTATCCTTTGAGGGGTTTATCGTTTCATCATTCATTCATTCATTTATCCCCCTTTTAATTAATTAACTCTTTCTTCCTTAATCACTCTCTCTAATCACTCATCACTGGGAAATTAATTAAATTAAATCTCTGTNGATGTAATTTAAAGAAAGAAAAAAGATTTTGTAAAAATGAGAACTTTATCGACGCCGGTGAAGTACTCTGTNAACAAAAGCGTNGTAAACAAGCGTNTCCCTCCTCCTTCTTCTNTCCCCTCTTCCTCAACTTGTCGTAAAGNGGTTCGATTTTCTGTCNGAGATGGCGACGCTACTGACTCCTCTGACGACGAATACGACGGCGTTTACATTCCCCGNGTTATTGACCATGTTTCCGAGATCCGTTATGAACCGGACCTTCCTCAGCCCGTGGCCCAGCCCAACAAGAATCCGGTCAGGGAACCGACTAAAACGACGACAGCAACCGAAAACGGTGAGGTTTTTAAGAAGTATCGTGGGGTCCGAC

## Supplementary File 2


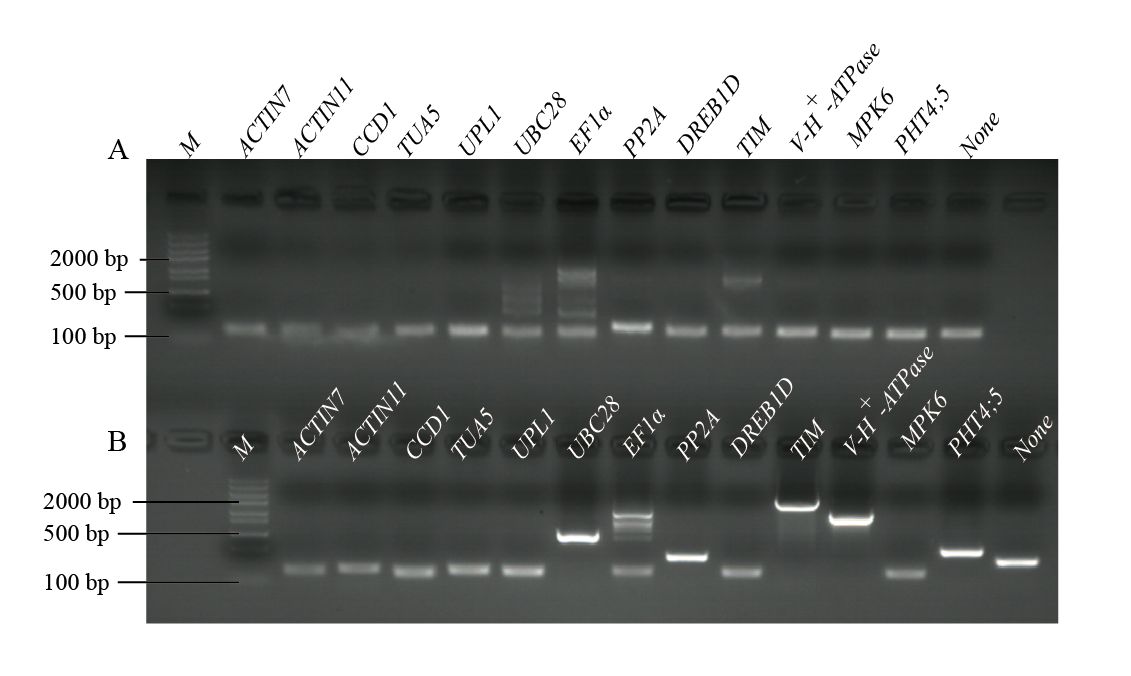


**Supplementary File 2:** PCR amplification specificity of the candidate reference genes using cDNA (A) and genomic DNA (B) as templates. The gel images come from the same gel. M: DL 5000 marker. The names of candidate reference genes were listed at the beginning of each lane. None: a candidate reference gene initially selected but not used in this study.

## Supplementary File 3


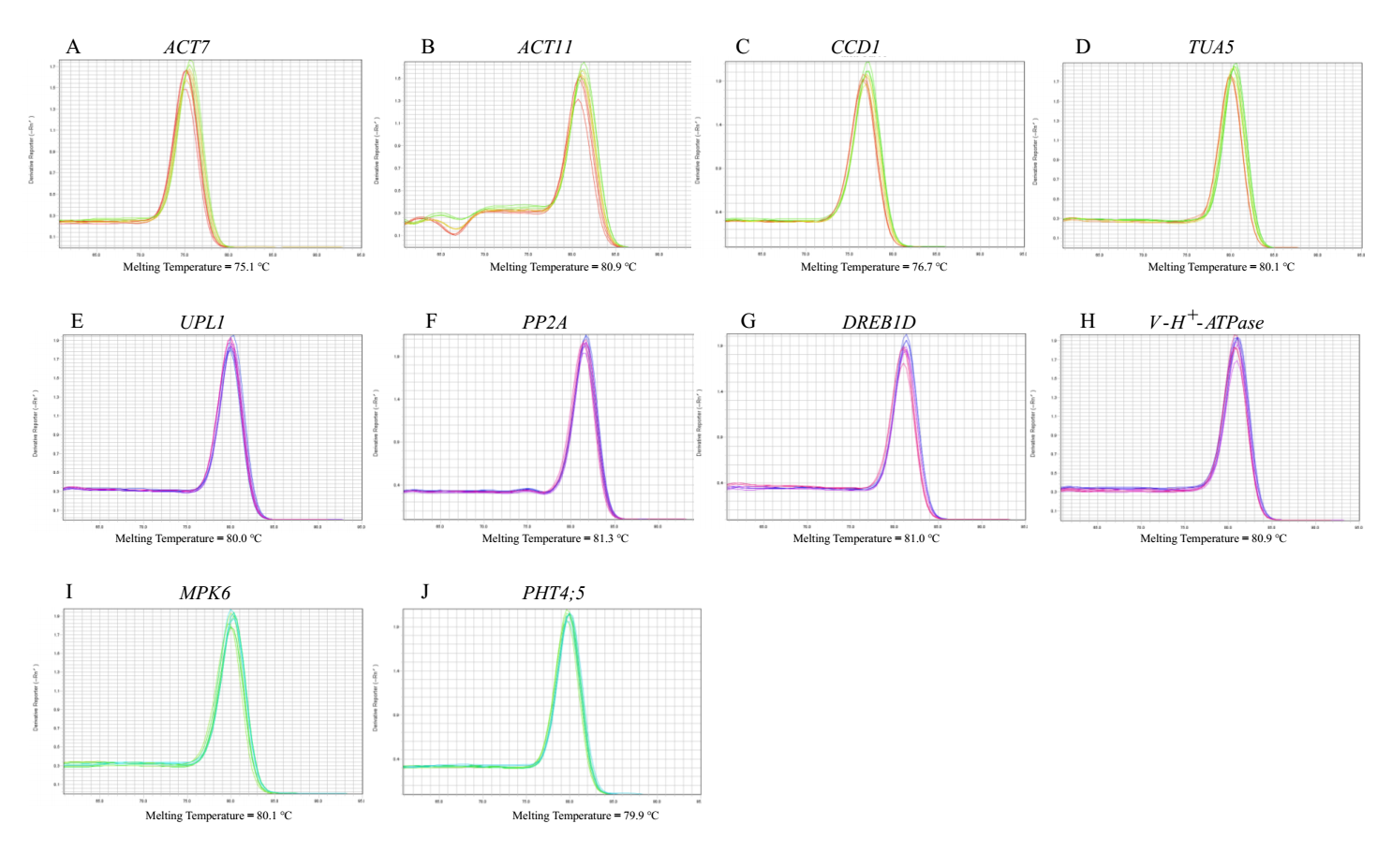


**Supplementary File 3:** PCR amplification specificity of the candidate reference genes identified by melting curves analysis. (A-J) Melting curves of *ACT7*, *ACT11*, *CCD1*, *TUA5*, *UPL1*, *PP2A*, *DREB1D*, *V-H^+^-ATPase*, *MPK6*, and *PHT4;5* of *S. glauca*.
